# TNF-α induces endothelial–mesenchymal transition promoting stromal development of pancreatic adenocarcinoma

**DOI:** 10.1038/s41419-021-03920-4

**Published:** 2021-06-25

**Authors:** Marjorie Adjuto-Saccone, Philippe Soubeyran, Julie Garcia, Stéphane Audebert, Luc Camoin, Marion Rubis, Julie Roques, Bernard Binétruy, Juan Lucio Iovanna, Roselyne Tournaire

**Affiliations:** 1 Centre de Recherche en Cancérologie de Marseille (CRCM), INSERM U1068, CNRS UMR 7258, Aix-Marseille Université and Institut Paoli-Calmettes, Parc Scientifique et Technologique de Luminy, Marseille, France; 2 Centre de Recherche en Cancérologie de Marseille (CRCM), INSERM U1068, CNRS UMR 7258, Marseille Protéomique, Aix-Marseille Université and Institut Paoli-Calmettes, Marseille, France; 3 INMED, INSERM U1249, Aix-Marseille Université, Parc Scientifique et Technologique de Luminy, Marseille, France

**Keywords:** Cancer microenvironment, Cell signalling

## Abstract

Endothelial–mesenchymal transition (EndMT) is an important source of cancer-associated fibroblasts (CAFs), which facilitates tumour progression. PDAC is characterised by abundant CAFs and tumour necrosis factor-α (TNF-α). Here, we show that TNF-α strongly induces human endothelial cells to undergo EndMT. Interestingly, TNF-α strongly downregulates the expression of the endothelial receptor TIE1, and reciprocally TIE1 overexpression partially prevents TNF-α-induced EndMT, suggesting that TNF-α acts, at least partially, through TIE1 regulation in this process. We also show that TNF-α-induced EndMT is reversible. Furthermore, TNF-α treatment of orthotopic mice resulted in an important increase in the stroma, including CAFs. Finally, secretome analysis identified TNFSF12, as a regulator that is also present in PDAC patients. With the aim of restoring normal angiogenesis and better access to drugs, our results support the development of therapies targeting CAFs or inducing the EndMT reversion process in PDAC.

## Introduction

Pancreatic ductal adenocarcinoma (PDAC) represents >90% of all pancreatic tumours. It is the fourth leading cause of cancer-related death, with 5-year survival rates remaining less than 8% [1–3]. Although the use of combined chemotherapies has improved responses, survival gains in patients remain limited [4–6], underlining the urgent requirement of new therapeutic targets to improve PDAC patient survival. PDAC is characterised by a desmoplastic stroma that represents 90% of the tumour and compresses blood vessels leading to hypovascularisation [4, 7] and poorly perfused tumours [8], thereby preventing efficient delivery of chemotherapeutic drugs [9].

Apart from the extracellular matrix (ECM), infiltrated immune cells and endothelial cells, the most abundant cells present in this tumour microenvironment (TME) are cancer-associated fibroblasts (CAFs) [10, 11]. CAFs are key components of PDAC because they facilitate tumour progression by secreting ECM and soluble factors that stimulate cancer progression, invasion and metastasis [10, 12]. CAFs are also responsible for suboptimal drug delivery to tumour cells and equally involved in drug resistance [12–15].

Zeisberg et al. demonstrated, in mouse models of cancer, that endothelial**–**mesenchymal transition (EndMT) is an important source of CAFs [16, 17]. We have previously demonstrated that EndMT occurs in human tumours [18]. EndMT, a form of epithelial**–**mesenchymal transition (EMT) [19–23], is a process in which cells lose their polarity and expression of endothelial markers and instead acquire mesenchymal markers and invasive and migratory properties [16–18, 22–24]. EndMT plays an important role in embryonic heart formation and is involved in a variety of tissue fibrosis [22, 25] and other uncommon diseases such as fibrodysplasia ossificans progressive (FOP) and cerebral cavernous malformation [26].

We investigate the factors that, in PDAC, induce EndMT and CAF production. We especially focus on the pro-inflammatory cytokine tumour necrosis factor-α (TNF-α) because it induces an acute and chronic inflammation directly linked to various steps involved in tumorigenesis [20, 27–29] and, explore the hypothesis that the TNF-α, abundantly present in PDAC, induces EndMT.

## Results

### TNF-α induces endothelial–mesenchymal transition

**Fig. 1:**
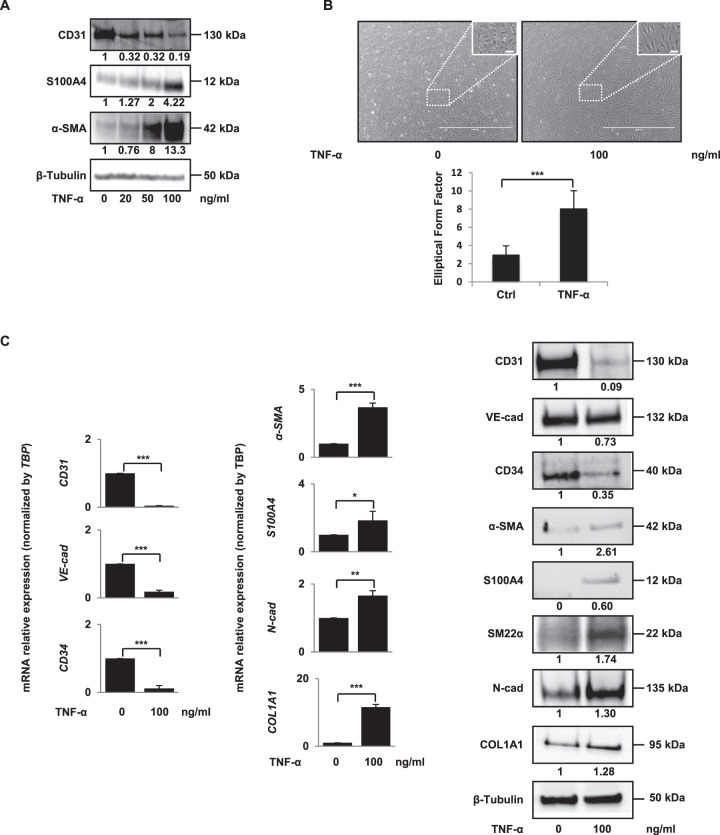
TNF-α induces endothelial–mesenchymal transition. **A** TNF-α decreases the protein expression of vascular endothelial marker (CD31) and increases the protein expression of mesenchymal markers (S100A4, α-SMA). HMVECs were treated for 96 h with 0, 20, 50 or 100 ng/ml of TNF-α. Protein expressions were analysed by western blot. **B** Morphological changes induced by a 100 ng/ml TNF-α treatment (photographs). The histogram shows ImageJ software analysis of cell morphology by calculating the elliptical form factor EFF (the major axis divided by minor axis). Results are the average of 100 cells. **C** TNF-α decreases the mRNA (left panel) and protein (right panel) expression of vascular endothelial markers (CD31, CD34, VE-cadherin) and increases the mRNA (left panel) and protein (right panel) expression of mesenchymal markers (COL1A1, N-cadherin, S100A4, α-SMA, SM22-α). mRNA levels were quantified by RT-qPCR. *TBP* for RT-qPCR and β-tubulin for western blot analysis were used as controls. RT-qPCR histograms show the mean of three independent biological experiments and western blots are representative of three independent biological experiments. Significant differences are indicated by solid lines (*P < 0.1, **P < 0.05, ***P < 0.005 by *t* test). Scale bars, 100 µm (inset) or 2000 µm. HMVEC human microvascular endothelial cell, TNF-α tumour necrosis factor-α, α-SMA α-smooth muscle actin, VE-cad VE-cadherin, COL1A1 collagen type I α1, N-Cad N-cadherin, Ctrl control, mRNA messenger RNA, *TBP* TATA-box binding protein.

Human microvascular endothelial cells (HMVECs) were treated with 20, 50 or 100 ng/ml of TNF-α added once (Fig. 1A) or daily for 4 days (Supplemental Fig. 1A). After 96 h, cells were lysed and protein expression was analysed by western blot. Results show that TNF-α reduced the protein expression of the vascular endothelial marker CD31 and reciprocally augmented the expression of the mesenchymal markers α-SMA and S100A4. Dose-dependent these effects were very strong already from 20 ng/ml of TNF-α. At 100 ng/ml, we observed a strong morphological change, from epithelioid to an elongated and spindle-shaped fibroblast-like appearance (Fig. 1B), suggesting an EndMT process. To quantify these morphological differences between TNF-α-treated and untreated cells, we measured the elliptical form factor EFF. Results show that TNF-α-treated cells are much more elongated than untreated cells (Fig. 1B). For further experiments, we chose the concentration of 100 ng/ml. Analysis of additional endothelial and mesenchymal markers confirmed these results (Fig. 1C). Furthermore, FACS analysis demonstrates that the TNF-α-induced decrease of CD31 protein expression affects a large majority of endothelial cells (Supplemental Fig. 1B).

Various incubation times were then tested (Supplemental Fig. 1C), showing that the downregulation of CD31 began at 24 h, the upregulation of α-SMA began at 48 h and that these effects were maintained up to at least 168 h.

In addition, we obtain similar results on primary cultures of endothelial cells: human umbilical vein endothelial cells (HUVECs) (Supplemental Fig. 1C), suggesting that these effects represent a systematic response of endothelial cells to TNF-α.

**Fig. 2:**
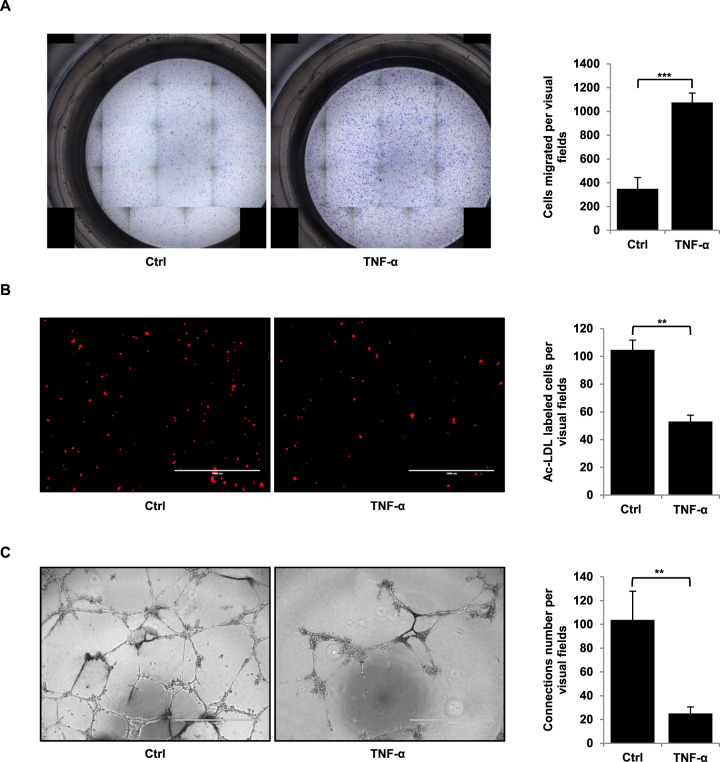
Effects of TNF-α on cellular migration and angiogenesis. **A** TNF-α increases HMVECs migration. HMVECs were treated or not for 96 h with 100 ng/ml of TNF-α and were allowed to migrate in a modified Boyden chamber assay for an additional 3 h30. **B** TNF-α decreases the number of Ac-LDL-labelled endothelial cells. **C** TNF-α induces a reduction in tubules formation in a matrigel-based tube-formation assay. Tube formation and Ac-LDL scores were measured for three fields. Scale bars, 1000 µm. Migration was measured for six fields. Similar results were obtained in three different experiments. Significant differences are indicated by solid lines (**P < 0.05, ***P < 0.005 by *t* test). HMVEC human microvascular endothelial cell, Ctrl control, TNF-α tumour necrosis factor-α.

During EndMT, endothelial cells acquire a mesenchymal phenotype characterised by the acquisition of invasive and migratory properties [17]. We thus tested the effects of TNF-α on endothelial cell migration. TNF-α is found to increase cell migration by more than threefold compared with untreated control (Fig. 2A).

We also tested the effects of TNF-α on angiogenesis using two angiogenic tests. Firstly, we incubated cells with TNF-α and Ac-LDL (acetylated low-density lipoprotein), an agent specifically metabolised by endothelial cells, thus allowing their identification (Fig. 2B). Results show firstly that TNF-α does not interfere with the proliferation of HMVEC cells (Supplemental Fig. 1D), and subsequently that Ac-LDL-labelled endothelial cells drastically decreased by 44% compared to untreated control. Secondly, we performed a Matrigel-based tube-formation assay to assess the vasculogenic activity of cells treated or not with TNF-α (Fig. 2C). Results show a dramatic reduction (76%) in the formation of tubules in the presence of TNF-α.

Therefore, following our investigations of various criteria, we can conclude unambiguously that TNF-α induces EndMT.

### TNF-α activates intracellular signalling pathways and TNF-α-induced EndMT is SNAI1-, SNAI2- and ZEB2-dependent

**Fig. 3:**
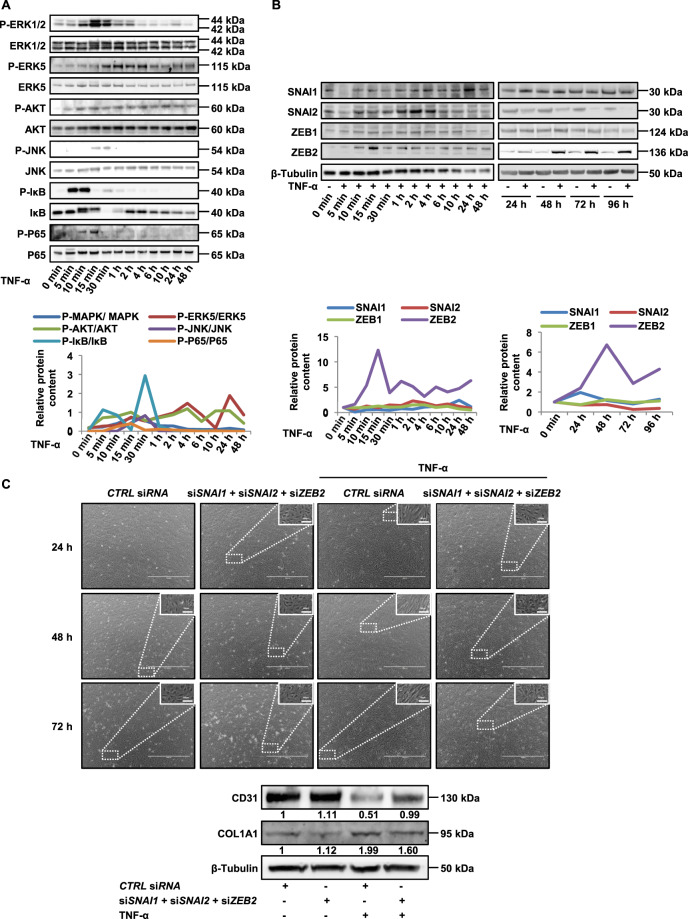
Effects of TNF-α on signal transduction. **A**, **B** TNF-α increases Erk1/2, Erk5, Akt, JNK, IkB and P65 phosphorylations (**A**) and protein expression of SNAI1, SNAI2 and ZEB2 (**B**) analysed by western blot. HMVECs were treated for various times with 100 ng/ml of TNF-α. Western blot quantifications are plotted on the graphs below. **C** *SNAI1*, *SNAI2* and *ZEB2* deficiencies partially prevented the morphological changes induced by TNF-α (upper panel) and abrogated or partially reverted the modifications induced by TNF-α on the expression of vascular endothelial marker (CD31) and mesenchymal markers (COL1A1) (lower panel). HMVECs were transfected with *SNAI1*, *SNAI2* and *ZEB2* or *CTRL* siRNA and treated for various times with 100 ng/ml of TNF-α. Total ERK1/2, ERK5, Akt, JNK, IkB, P65 and β-tubulin were used as controls for western blots. Data are representative of three independent experiments. Scale bars, 100 µm (inset) or 1000 µm. HMVEC human microvascular endothelial cell, TNF-α tumour necrosis factor-α, Erk1/2, 5 extracellular signal-regulated kinase 1/2, 5, COL1A1 collagen type I α1, Ctrl control, siRNA short-interfering RNA.

To get insights in the molecular mechanisms involved in this TNF-α-induced EndMT, we investigated signalling pathways and transcription factors known to be involved in EndMT. We focus on PI3K/Akt, Erk1/2 and Erk5 pathways because they are upregulated by the activation of EMT or EndMT in cells [18, 22]. Moreover, JNK and NFκB pathways are known to be activated by TNF-α [30, 31]. As shown in Fig. 3A, compared with control, TNF-α treatment increased Erk1/2, Erk5, Akt, JNK, IκB and p65 phosphorylation with slight kinetic differences (Fig. 3A), Akt, ERK5 and IκB being activated as early as 5 min, whereas the other pathways are activated only after 10–15 min. Altogether these results show that all these pathways are induced by TNF-α during EndMT, suggesting a potential role in this process.

It is known that EndMT and EMT activation by signalling pathways leads to an increased expression of the *SNAIL* and *ZEB* gene families of transcription factors [17]. We investigated whether the expression of these transcriptional factors was altered by TNF-α during EndMT. SNAI1 mRNA is induced as early as 15 min after TNF-α stimulation, while its protein appears upregulated only after 24 h, suggesting a complex post-transcriptional control (Fig. 3B and Supplemental Fig. 2A). SNAI2 mRNA and protein are induced as 5 min and after 2 h, respectively, whereas ZEB1 expression shows no regulation. Interestingly, ZEB2 expression shows induction of its protein already after 15 min of stimulation, whereas its mRNA is induced only after 2 h, suggesting the hypothesis of a dual effect of TNF-α on ZEB2 expression, a first rapid effect on ZEB2 protein stability, followed later on by a transcriptional stimulatory effect. Altogether, these results show that SNAI1, SNAI2 and ZEB2 transcription factors are activated after TNF-α stimulation.

To investigate the role of these transcription factors in the TNF-α effect, we transfected cells with *SNAI1*, *SNAI2* and *ZEB2* siRNAs. We found a decrease in the corresponding expression of these genes in TNF-α-treated cells (Supplemental Fig. 2B) and importantly the TNF-α stimulatory effect was almost abolished (Fig. 3C). Transfected independently none of the siRNAs was able to inhibit TNF-α-induced EndMT (not shown), however concomitant transfection of *SNAI1*, *SNAI2* or *ZEB2* siRNAs, partially prevented the morphological changes induced by TNF-α (Fig. 3C, top), strongly increased CD31 expression and repressed mesenchymal expression of COL1A1 (Fig. 3C, bottom).

### TIE1 is implicated in TNF-α-induced EndMT

Mechanistically, we have shown previously that EndMT is mediated by deficiency of the receptor tyrosine kinase TIE1 in human endothelial cells [18]. TIE1 is an endothelial receptor essential for the development and maintenance of the vascular system [32].

**Fig. 4:**
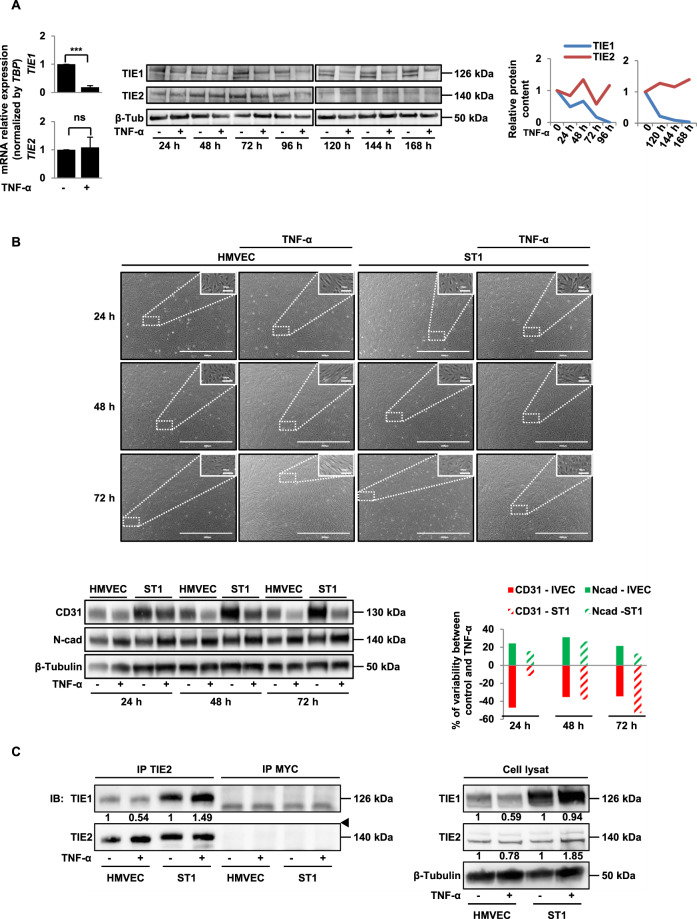
Effects of TNF-α on TIE1 and TIE2 receptors. **A** TNF-α decreases the mRNA and protein expression of TIE1 and had no effect on TIE2 expression. HMVECs were treated various times with 100 ng/ml of TNF-α. mRNA and protein levels were quantified by RT-qPCR and western blot, respectively; graphs represent protein quantifications. RT-qPCR histograms show the mean of three independent biological experiments, and western blots are representative of three independent biological experiments. **B**, **C** The overexpression of TIE1 induces a delay of TNF-α-induced EndMT (**B**) and increases TIE1/TIE2 interactions (**C**). Control HMVECs and *TIE1*-encoding lentivirus infected clone (ST1) were treated various times with 100 ng/ml of TNF-α. Scale bars, 100 µm (inset) or 2000 µm. The graph represents the variation rate of proteins in presence of TNF-α. Lysates from control HMVECs and overexpressing TIE1 ST1 cells were subjected to immunoprecipitation with an anti-TIE2 or anti-MYC antibody, and TIE1 was detected by western blot. The arrow shows an aspecific band. *TBP* for RT-qPCR and β-tubulin for western blot analysis were used as controls. Data are representative of three independent experiments. Significant differences are indicated by solid lines (***P < 0.005 by *t* test). HMVEC human microvascular endothelial cell, EndMT endothelial–mesenchymal transition, TNF-α tumour necrosis factor-α, ST1 cells overexpressing TIE1, N-Cad N-cadherin, Ctrl control, mRNA messenger RNA, TBP TATA-box binding protein.

Therefore, we hypothesised that TNF-α-induced EndMT could depend on TIE1 regulation. We found that TNF-α decreases the mRNA and protein expression of TIE1 (Fig. 4A), whereas it has no effect on TIE2 expression, the other member of the TIE receptor family [33] (Fig. 4A). FACS analyses showed that the TNF-α-induced decrease of TIE1 expression concerns all endothelial cells (Supplemental Fig. 3A). To characterise whether TNF-α acts on *TIE1* transcription or degradation, we incubated HMVECs with TNF-α in the presence or absence of actinomycin D. Results show that in the presence of actinomycin D and TNF-α, the decrease in *TIE1* mRNA is greater than with actinomycin D or TNF-α alone (Supplemental Fig. 3B), suggesting that TNF-α acts mainly by regulating its TIE1 half-life rather than its transcription. In order to determine whether *TIE1* deficiency is involved in the induction of EndMT by TNF-α, we stably overexpressed TIE1 in HMVECs using a *TIE1* lentivirus vector. Overexpression of TIE1 was obtained in three independent cell clones (Supplemental Fig. 3C). Remarkably, compared to TNF-α-treated control HMVECs, we observed a delay in the occurrence of EndMT-related morphological features in TNF-α-treated cells overexpressing TIE1 (ST1) (Fig. 4B). This delay was also observed by the analysis of EndMT markers at 24 h; whereas TNF-α induces a 47% decrease in CD31 and a 24.2% increase in N-cadherin in HMVECs, signing the EndMT, this effect is much lower in ST1 cells with only 11% decrease in CD31 and 15.7% increase in N-cadherin (Fig. 4B). Nevertheless, overexpression of TIE1 did not completely prevent TNF-α-induced EndMT (Fig. 4B).

It has been shown that TIE1 modulates TIE2 signalling by forming heterodimers [34, 35]. We, therefore, studied the effects of TNF-α on TIE1/TIE2 heterodimers in HMVECs and ST1 cells (Fig. 4C). Co-immunoprecipitation experiments revealed that TIE1 co-precipitates with TIE2 in HMVECs. In agreement with the TNF-α-induced decrease in TIE1 expression, TIE1/TIE2 heterodimers are decreased in TNF-α-treated HMVECs. In contrast, in ST1 cells, TNF-α has no effect on ectopic TIE1 expression, probably due to the lack of TNF-α-targeted regulatory sequences in the lentivirus vector, and we found that TNF-α induced a 50% increase in TIE1/TIE2 interactions in ST1 cells. Altogether our results suggest a role for TIE1/TIE2 heterodimers in TNF-α-induced EndMT.

### TNF-α-induced EndMT is reversible

Reversing the CAF phenotype may provide a strategy for developing therapeutic approaches to prevent fibrotic complications and increase vascularity in tumours. MET, the reverse process of EMT [19, 20, 22, 23], has been widely described during embryogenesis. Furthermore, MET is recognised as critical for the later stages of tumour metastasis by allowing cancerous cells to regain epithelial properties and integrate into distant organs [36].

**Fig. 5:**
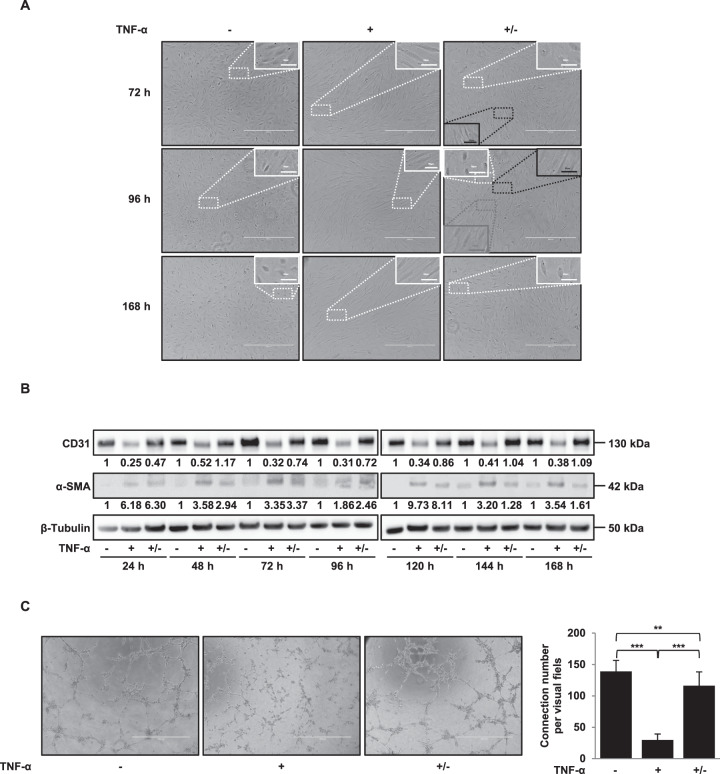
TNF-α-induced EndMT is reversible. **A**, **B** TNF-α removal reverses the spindle-shaped phenotype induces by TNF-α (**A**) and TNF-α removal reverses the CD31 marker downregulation induced by TNF-α, restoring CD31 levels present in untreated controls (**B**). Reciprocal reversion is observed for the expression of the mesenchymal marker α-SMA. HMVECs were treated or not (−) for 72 h with 100 ng/ml of TNF-α then, cells were washed (+/−TNF-α) or not (+TNF-α) and cultured for various times. Scale bars, 50 µm (inset) or 400 µm. Protein expressions were analysed by western blot. **C** TNF-α induces a reduction in the formation of tubules in a matrigel-based tube-formation assay (+TNF-α), and a tubule restoration is observed from 168 h after removal of TNF-α (+/−TNF-α). Scale bars, 1000 µm. β-tubulin was used as loading Ctrl for western blot analysis. Tube-formation scores were measured for three fields. Similar results were obtained in three different experiments. Significant differences are indicated by solid lines (**P < 0.05, ***P < 0.005 by *t* test). HMVEC human microvascular endothelial cell, TNF-α tumour necrosis factor-α, α-SMA α-smooth muscle actin, Ctrl control.

In contrast to MET, MEndT (mesenchymal**–**endothelial transition) is very poorly described. We investigated potential MEndT by removing TNF-α after TNF-α-induced EndMT. HMVECs were incubated with TNF-α for 72 h, then cells were washed (+/− TNF-α) or not (+ TNF-α) and cultured for various times. Morphological changes were analysed at various time points (Fig. 5A and Supplemental Fig. 4A). Whereas all cells display a fibroblast cell-like phenotype in + TNF-α cultures at any time points, we observed two types of cell populations 72 h after TNF-α removal in + /− TNF-α cultures: fibroblast cell-like phenotypes and a few endothelial cell-like phenotypes. At 96 h, we observed three populations in + /− TNF-α cultures: fibroblast cell-like phenotypes, endothelial/fibroblast cell-like phenotypes and endothelial cell-like phenotypes; finally, at 168 h, we observed only endothelial cell-like phenotypes. Figure 5B shows that the strong decrease in CD31 expression following TNF-α treatment was reversed 24 h after TNF-α removal; the expression of CD31 was identical to that found in the untreated control. FACS analysis confirmed that the whole cell population re-expresses CD31 at 168 h after removal of TNF-α (Supplemental Fig. 4B). Downregulation of α-SMA in + /− TNF-α cultures is found from 48 h, delayed as compared to CD31 regulation found already from 24 h (Fig. 5B). This result could explain the persistence of fibroblasts-like phenotypes at early time points in + /− TNF-α cultures (Fig. 5A and Supplemental Fig. 4A). This reversion persisted until at least 168 h after removing TNF-α. We then examined the effects of TNF-α removal on the formation of tubules in a Matrigel-based tube-formation assay. A tubule restoration capacity was observed 72 h after TNF-α removal (Fig. 5C).

Altogether our results show that fibroblasts undergo MEndT to generate de novo endothelial cells.

### Analysis of the protein secretome induced by TNF-α

**Fig. 6:**
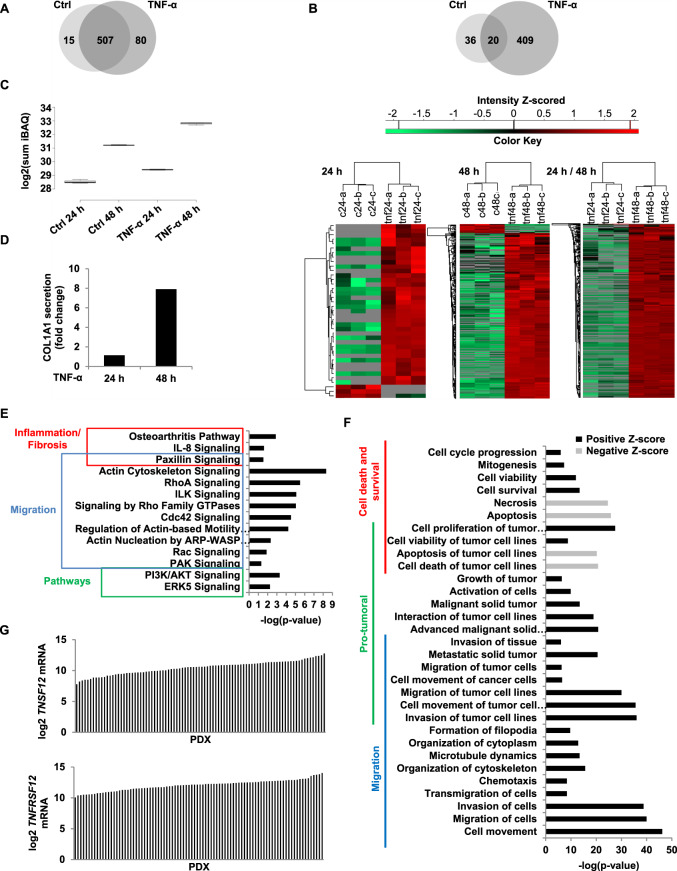
Analysis of HMVECs secretome induced by TNF-α. HMVECs were treated for 24 h and 48 h with 100 ng/ml of TNF-α and supernatants were analysed by mass spectrometry. **A** The Venn diagram presents proteins detected in HMVECs secretome treated or not with TNF-α. In total, 602 proteins were detected whose 15 only in HMVECs secretome, 80 only in HMVEC’s secretome treated with TNF-α and 507 were common to both. **B** The Venn diagram depicting total protein distribution, 20 proteins were not significantly different between subpopulations, 36 and 409 were significantly elevated in the endothelial and mesenchymal subpopulations, respectively, using a 1.5 cut-off for significant differences. Heatmap of proteins differentially expressed between Ctrl and TNF-α at 24 h and 48 h and between TNF-α 24 h and TNF-α 48 h. Results were obtained with three technical replicates. **C**, **D** TNF-α induces an increase of secretory capacity of HMVECs as revealed by the sum of abundance intensity (iBAQ) for all identified proteins (**C**) and an increase of COL1A1 secretion (**D**). **E**, **F** All proteins detected at 48 h of TNF-α treatment were analysed with IPA. Pathways (**E**) and functions (**F**) significantly affected are represented by the graphs. **G** mRNA expression levels of *TNSF12* and *TNFRSF12* in PDXs. HMVECs human microvascular endothelial cells, TNF-α tumour necrosis factor-α, Ctrl control, COL1A1 collagen type I α1, *TNFSF12* tumour necrosis factor ligand superfamily member 12, *TNFRSF12* tumour necrosis factor receptor superfamily member 12, PDXs patient-derived xenografts.

CAFs are known to have important secretory capacities promoting tumour progression [37]. In order to find out whether TNF-α modifies the secretory capacities of treated cells, we performed a comparative analysis of HMVEC secretomes treated or not treated with TNF-α for 24 and 48 h (Fig. 6).

Mass spectrometry led to the identification of 602 secreted proteins at 48 h, among which 15 were specific to untreated and 80 were specific to treated cells (Fig. 6A). A MaxQuant analysis was performed to determine differentially expressed proteins. At 48 h, only 20 proteins showed no significant differences between the two secretomes (Fig. 6B and Supplemental Fig. 5B). These results show an important specific secretion by treated cells. Next, secretory capacity was determined using the intensity-based absolute quantification (iBAQ). Compared with control at 24 h and 48 h, TNF-α induced a 1.9- and threefold increase in secreted proteins, respectively (Fig. 6C). This increase is also directly detected on PAGE gel (Supplemental Fig. 5A).

CAFs are characterised by their pro-inflammatory signature [38] and by the production of high levels of collagen [39]. Table 1 in Supplemental Fig. 5C shows that pro-inflammatory proteins are upregulated in the secretome of treated cells. Likewise, cells treated with TNF-α for 48 h produce 7.9-fold more Col1a1 than untreated cells (Fig. 6D). These results are in agreement with the fibroblastic features of TNF-α-treated endothelial cells.

We analysed all the secreted proteins detected at 48 h of treatment using the Ingenuity Pathway Analysis (IPA) software. Among pathways significantly affected by the changes in protein secretion and predicted to be activated, several are implicated in migration, inflammation and fibrosis, supporting the fibroblastic nature of treated cells (Fig. 6E). In agreement with our results (Figs. 3A and 2A), AKT and ERK5 pathways are predicted to be activated in treated cells (Fig. 6E), as well as processes related to migration (Fig. 6F). It is known that TNF-α can induce apoptosis, necrosis or invasion of tumoural cells. Interestingly, necrosis and apoptosis pathways are, on the contrary, inhibited in our assay, while cell survival, viability and cycle progression pathways are activated (Fig. 6F). Furthermore, Fig. 6F shows 16 pro-tumoural processes activated. We conclude that TNF-α-treated endothelial cells displayed the pro-tumoural characteristics of CAFs. Table 2 in Supplemental Fig. 5C presents the regulators of signal transduction and inflammation predicted to be activated. Confirming the results presented in Fig. 3, the NFκB complex, JNK, ERK and PI3K are predicted to be activated. Among the activated upstream regulators, we focused our attention on TNFSF12 (tumour necrosis factor ligand superfamily member 12) (Table 2 in Supplemental Fig. 5C). Indeed, excessive activation of the TNFSF12 receptor pathway has been described as promoting pathological fibrosis in chronic liver disease by inducing the proliferation of hepatic stellate cells [40, 41]. In order to determine whether TNFSF12 and its receptor were present in PDAC patients, PDX (patient-derived xenografts) were analysed. Results obtained by RNA-seq demonstrate that tumours from all patients examined exhibited high levels of *TNFSF12* and *TNFRSF12* (Fig. 6G), suggesting the activation of this pathway and pointing at its importance in PDAC.

### TNF-α induces CAFs in pancreatic tumours in vivo

We examined whether TNF-α induces the formation of CAFs and fibrosis in pancreatic tumours and thereby whether TNF-α may be responsible for the strong desmoplastic reaction characteristic of PDAC. PK4A mouse pancreatic tumour cells from spontaneous tumours were implanted orthotopically in syngeneic *Ink4a/Arf*^*fl/fl*^; *LSL-Kras*^G12D^ transgenic mice. The following day, we began injecting TNF-α every day for 4 weeks and analysed the impact of TNF-α on stroma organisation. Collagen fibres and fibrosis were revealed by Masson’s trichrome (MT) staining.

**Fig. 7:**
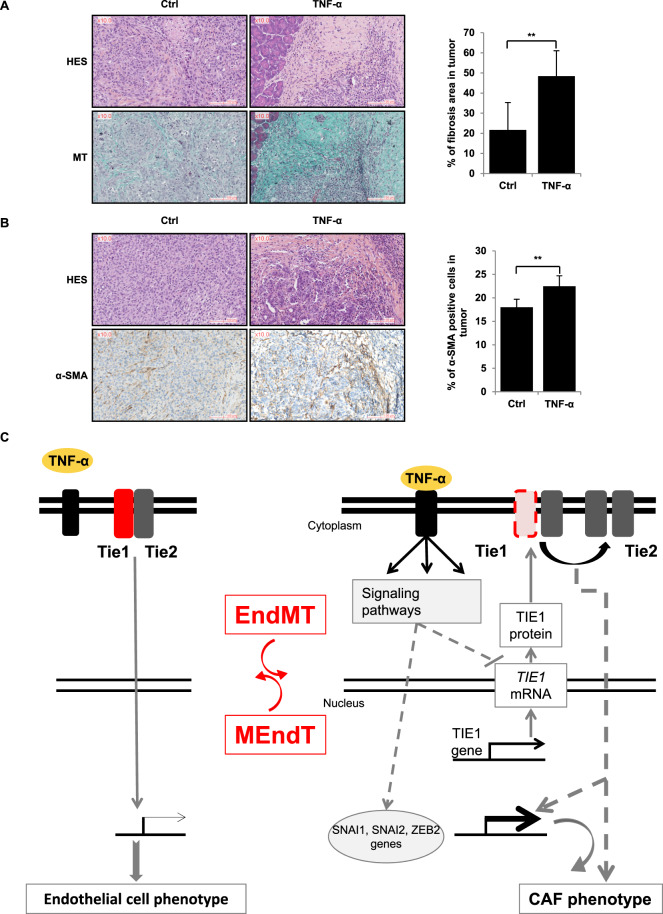
In vivo effect of TNF-α and schematic model of its action. TNF-α increases CAFs in pancreatic tumour in vivo (**A**, **B**) and schematic model of the reversible TNF-α-induced EndMT involving Tie1 downregulation (**C**). **A**, **B**. TNF-α induces an increase of fibrosis (**A**) and an increase of CAFs α-SMA positives (**B**). PK4A tumoral cells were orthotopically injected in mice which were treated daily for 27 days with injections of NaCl (Ctrl) or TNF-α (*n* = 6 for each condition). Then, pancreases were collected and sections of tissue were subjected to HES staining, Masson’s trichrome (MT) staining and α-SMA staining. Fibrosis and α-SMA staining were analysed and quantified by calopix. Significant differences are indicated by solid lines (**P < 0.05, by *t* test). Scale bars, 100 µm. CAF cancer-associated fibroblast, TNF-α tumour necrosis factor-α, Ctrl control, HES haematoxylin eosin saffron, MT Masson’s trichrome MT, α-SMA α-smooth muscle actin.

In agreement with the known elevated TNF-α secretion of pancreatic cells, collagen fibrosis (bright blue staining) was observed in tumour sections of untreated mice, however, quantification of the intensity of the staining revealed that TNF-α-treated mice present a twofold increase in fibrosis compared with untreated mice (Fig. 7A).

The activated fibroblast (myofibroblast or CAF) marker α-SMA [42, 43] was also quantified in both groups. We found that the percentage of α-SMA-positive cells was significantly (*P* < 0.05) higher in TNF-α-treated mice (Fig. 7B), possibly reflecting an increase in the number of EndMT-derived fibroblasts. Importantly, these results indicate that TNF-α induces an increase in CAFs also in vivo in murine pancreatic tumours.

## Discussion

CAFs are one of the most crucial components of the microenvironment which promotes tumour growth and invasion [10, 12]. Furthermore, the abundant fibrotic stroma produced by CAFs constitutes a physical barrier against effective drug delivery, contributing strongly to chemoresistance in pancreatic cancer [7].

We show that TNF-α, abundantly present in PDAC, induces EndMT, acts, at least partially, through TIE1 regulation and participates in the CAF generation process in murine pancreatic tumours. In addition, secretome analysis identified TNFSF12 as a regulator, which is present in all PDAC of PDX.

We first show here that TNF-α induces the phenotypic conversion of endothelial cells into fibroblasts. We observed the reduction of vascular endothelial markers and the expression of mesenchymal markers [44]. In accordance with previous studies, this EndMT is accompanied by an increase in cell migration [18].

We find that TNF-α also increases SNAI1, SNAI2 and ZEB2 mRNA and protein expression. These transcription factors are known to repress the expression of VE-cadherin [45]. They very probably act synergistically, since several studies suggest that they regulate each other. Indeed, SNAI1 can upregulate *ZEB1* and *ZEB2* [46] and SNAI2 indirectly upregulates *SNAI1* [47]. All this is in agreement with our results, where *SNAI1*, *SNAI2* and *ZEB2* deficiencies partially prevented TNF-α-induced EndMT. This partial inhibition of the TNF-α effect could be due to residual expression of the transcription factors or, alternatively, to other transcription factors implicated in TNF-α-induced EndMT.

Several studies show that either vascular endothelial growth factor (VEGF) overexpression [49] or inflammatory molecules specific to PDAC [44, 48] induce a decrease in TIE1 protein by activating its ectodomain cleavage. Here, we found that TNF-α downregulates TIE1 by degrading *TIE1* mRNA, revealing an unknown TIE1 targeting molecular mechanism. Therefore, TNF-α can be expected to act on both *TIE1* mRNA and TIE1 protein cleavage. We show that overexpression of TIE1 partially prevents EndMT, these results suggest that in addition to TIE1 downregulation, TNF-induced EndMT necessitates the activation of additional pathways.

With regard to the receptor TIE2, we show that TNF-α does not change TIE2 expression, contrasting with the strong decrease in TIE1 expression. This result is in agreement with our previous results showing that, in PDAC, TIE2 expression persists in cells that had undergone EndMT in the absence of TIE1 [18]. These results are also in accordance with previous study in FOP showing that TIE2 does not decrease when endothelial cells become mesenchymal following EndMT [24]. Consequently to the TNF-α-induced diminution of Tie1 expression, which leads to the decrease in the proportion of TIE1/TIE2 heterodimers versus TIE2/TIE2 homodimers, our results show that there is a correlation between EndMT induction and TIE1/TIE2 heterodimers. Indeed, forced TIE1 overexpression in ST1 cells leads to a heterodimer increase and to a delay of EndMT after TNF-α treatment. However, we cannot exclude that this is the homodimer level that is important for TNF-α-induced EndMT. Further works are required to discriminate between these two hypotheses. Altogether our results are schematically summarised in the model presented in Fig. 7C. TNF-α has two types of receptors, TNFR1 (tumour necrosis factor receptor 1 or p55), expressed ubiquitously, and TNFR2 (tumour necrosis factor receptor 2 or p75), mainly expressed by immune and endothelial cells. In order to know which TNF-α receptors are involved during TNF-α-induced EndMT, it would be interesting to study the expressions of these receptors and to use neutralising antibodies or siRNA directed against them.

The desmoplastic and hypovascular nature of PDAC strongly contribute to its therapeutic resistance [12, 14, 49]. EndMT is very likely involved in the fact that PDAC are hypovascularized, with an 80% reduction in microvascular density compared with normal pancreases [48]. A loss of endothelial cells due to EndMT may contribute to microvascular rarefaction. To increase drug delivery, several approaches can be considered. Vessel normalisation is emerging as an opportunity to improve the efficacy of anti-angiogenic therapies [50–52], which can also transiently “normalise” the abnormal vessels [9, 53, 54]. In PDAC, the strategy of reversing CAF in endothelial cells seems more promising because of the highly desmoplastic nature of the tumour. Here, we show that EndMT can be reversed, with MEndT occurring rapidly after removing TNF-α. MEndT has been very poorly described. Ubil et al. demonstrated that cardiac fibroblasts undergo MEndT to generate endothelial cells in the injured heart and showed that MEndT can be augmented to contribute to neovascularization and enhance cardiac repair [55]. These exciting findings reveal a novel mechanism for new blood vessel formation: MEndT could contribute to neovascularization in PDAC and represents a potential therapeutic target for enhancing drug delivery.

Interestingly, anti-TNF-α therapy is currently used for inflammatory diseases as rheumatoid arthritis, and other pathologies. Accordingly to our results, previous study shows that inhibition of TNF-α by anti-TNF-α antibodies reduces both growth of PDAC tumours in PDX model and the number of pancreatic stellate cells (PSCs) as well as the amount of collagen [56, 57], indicating the possible effects of anti-TNF-α on PDAC-induced desmoplasia. However numerous studies suggest that anti-TNF-α treatments increase the risk for cancer [58], disqualifying this approach for PDAC. These apparent discrepancies could be due to the known pleiotropic downstream effects of TNF-α, and the dual nature of TNF-α which is known to be pro or anti-tumoural. In view of our results, the EndMT reversion could be achieved with greater specificity by targeting the TIE1 and TIE2 receptors. As we show here, the balance between the TIE2/TIE2 homodimers and TIE1/TIE2 heterodimers seem critical in this process and interfering with this balance should therefore regulate the EndMT reversion. In the end, one possibility for reversing the EndMT would be to act at the level of the TIE1/TIE2 heterodimers. In this respect, the very recently ligand of TIE1 (LECT2), identified in a different cellular context, could play a role in the PDAC [59]. One therapeutical approach could be to find peptides that inhibit LECT2/TIE1 binding by the Phage Display technique [50, 51] to block the inhibition of TIE1/TIE2 heterodimers.

We report in this paper that TNF-α induces EndMT, partially through TIE1 downregulation and that a MEndT is obtained by removing TNF-α. We show that in vivo administration of TNF-α strongly increases the formation of CAFs and an abundant desmoplastic stroma in pancreatic tumours. Our results unravel some of the EndMT mechanisms involved in tumour development and have implications for therapeutic targeting of EndMT or for therapies inducing a reversible process known as MEndT. Such therapeutic approaches should restore normal angiogenesis and, consequently, favour better access to drugs and diminish drug resistance.

## Methods

### Cell lines

HMVEC cell lines were kindly supplied by Dr Xing Guo (Duke University Medical Center, Durham, NC, USA) and were cultured according to Shao and Guo [60]. Cells were cultured in MCDB131 (Gibco) medium supplemented with 12% HyClone FBS (Healthcare Life Sciences), 10 mM glutamine (Invitrogen), 100 mg/ml heparin, 10 ng/ml FGF2 (Sigma), 10 ng/ml EGF (Corning), 1 μg/ml hydroxycortisone (Sigma). HMVECs were used until passage 20. Primary HUVEC (pHUVEC) were purchased (Gibco) and were cultured in Medium 200 supplemented with Low Serum Growth Supplement kit (LSGS, Gibco). pHUVECs were used until passage 5. PK4A cell lines were established from *Pdx1-Cre*; *LSL-Kras*^*G12D*^; *Ink4a/Arf*^*fl/fl*^ tumours [61] and were cultivated in Glutamax 25 mM glucose DMEM (Gibco) medium supplemented with 10% FBS (Biosera), 1% antibiotic/antimycotic (anti/anti). PK4A were used at passage 23.

### TNF-α treatment

In total, 1 × 10^6^ cells were seeded into 100-mm dishes. Twenty-four hours later, cells were washed twice with PBS and treated with human recombinant TNF-α (100 ng/ml; Sigma). For the α-SMA expression studies, cells were cultivated in medium without hydrocortisone and FGF2 because these compounds inhibit the expression of α-SMA [62].

### Signalling pathways

In total, 3 × 10^6^ cells were seeded into 100-mm dishes. When they adhered, cells were washed twice with PBS and cultivated in depleted medium (MCDB131; 1% glutamine). The next day, cells were washed twice, cultivated in depleted medium and treated with TNF-α (100 ng/ml; Sigma).

### RNA interference

*SNAI1*, *SNAI2* and *ZEB2* siRNAs were purchased from Eurogentec. Control siRNA was purchased from Eurofins (Supplemental Table 3). A non-relevant siRNA (Ctrl). Cells were transfected with siRNAs using the calcium phosphate method into 100-mm dishes containing 1 × 10^6^ HMVECs. After 6 h of transfection, cells were washed and the day after a new transfection was performed. After 48 h, transfected cells were treated for 48 h or 72 h with TNF-α (100 ng/ml; Sigma) and analysed as described hereunder.

### Quantitative real-time PCR

Total RNA was extracted using TRIzol (Invitrogen) and cDNAs were prepared using GoSCRIPT kit (Promega, Madison, WI, USA) following the manufacturer’s instructions. Quantitative PCR was performed in an AriaMx^®^ real-time PCR system (Agilent Technologies, Santa Clara, CA, USA), using the GoTaq^®^ qPCR Master Mix kit (Promega, Madison, WI, USA). Quantisation was performed using the Agilent AriaMx 1.6 software (Agilent Technologies, Santa Clara, CA, USA). Primer sequences are available in Supplemental Table 4.

### Western blot analysis

Cells were lysed in RIPA buffer (Sigma) supplemented with an inhibitor cocktail. Equal amounts of protein were submitted to SDS-PAGE (10%, 7%, 4–12% or 3–8% NuPAGE Novex Mini Gels, Invitrogen), and electrophoretically transferred onto polyvinylidene difluoride membrane (Immobilon-P). Membranes were blocked in Tris-buffered saline (10 mM Tris–HCl, pH 7.6, 0.15 M NaCl) containing 0.5% non-fat milk (or 1% bovine serum albumin) and 0.1% Tween-20 for 1 h at room temperature. Membranes were probed using the SNAPid system with either anti-TIE1 antibody, anti-TIE2 antibody, anti-VE-cadherin antibody, anti-P-Tyr antibody, anti-ZEB1 antibody (Santa Cruz Biotechnology), anti-CD34 antibody, anti-SM22α antibody, anti-col1a1 antibody (Abcam), anti-ERK1/2 antibody, anti-p-ERK1/2 antibody, anti-ZEB2 antibody (Sigma) or anti-CD31 antibody, anti-α-SMA antibody, anti-S100A4 antibody, anti-N-cadherin antibody, anti-SNAI1 antibody, anti-SNAI2 antibody, anti-ERK5 antibody, anti-p-ERK5 antibody, anti-AKT antibody, anti-p-AKT antibody, anti-JNK antibody, anti-p-JNK antibody, anti-IκB antibody, anti-p-IκB antibody, anti-P65 antibody, anti-p-P65 antibody (Cell Signaling Technology). An anti-β-tubulin antibody was used as a loading control. Immunoreactive bands (peroxidase activity) were visualised with the ECL system (Amersham Pharmacia Biotech) and analysed using a Fusion FX7 imager (Fisher Bioblocks Scientific).

### Cell migration assay

Endothelial cell migration assays were performed using a 24-well chemotaxis chamber (Transwell, Falcon) as described [63]. Polycarbonate Membranes with a pore size of 8 μm were coated with 50 μg/ml fibronectin (Sigma) and 0.1% gelatin in phosphate-buffered saline (PBS) overnight. Cells were harvested by trypsinization, washed three times with serum-free MCDB131 medium containing 0.1% fatty acid-free BSA and were seeded at 2 × 10^5^ cells per well. The lower wells contained serum-free medium with 0.1% BSA in the presence or absence of platelet-derived growth factor (PDGF)-BB (20 ng/ml; AbCys S.A.). The cell suspension was added to the upper chamber, and cells were allowed to migrate at 37 °C in a 5% CO_2_ humidified incubator. After 3 h, the filter was removed, and the upper side of the filter containing the non-migrated cells was scraped with a rubber policeman. The filters were fixed with methanol and stained with coomassie blue. Migration was quantified by counting cells in six random high-power fields in each well.

### Tubule-formation assay

Endothelial cell tubule-formation assays were performed using a 96-well plate. Wells were coated with Matrigel (Corning) 3 h at 4 °C. In total, 2 × 10^4^ endothelial cells, treated or not with TNF-α (100 ng/ml; Sigma) during 96 h, were seeded per wells in a medium with 0.5% FCS (Hyclone) and without FGF2 and hydrocortisone. Cells were allowed to form tubules at 37 °C in a 5% CO_2_ humidified incubator. After 13 h, each well is observed with an EVOS microscope.

### Low-density lipoprotein (LDL) assay

Low-density lipoprotein assays were performed using a 24-well plate. In total, 5 × 10^4^ endothelial cells, treated or not with TNF-α (100 ng/ml; Sigma) during 96 h, were seeded in a medium with 0.5% FBS (Hyclone) and without FGF2 and hydrocortisone. After cell adhesion, LDL (20 µg/ml, Life Technology) were added to each well and the plate was incubated at 37 °C in a 5% CO_2_ humidified incubator. After 13 h, each well is observed with an EVOS microscope.

### Actinomycin D assay

In total, 1 × 10^6^ cells were seeded into 60-mm dished. Twenty-four hours later, cells were washed twice, cultivated in medium without FGF2 and hydrocortisone and treated with Actinomycine D (5 µg/ml; Sigma). After 2 h, cells were treated with TNF-α (100 ng/ml; Sigma) and analysed as described above.

### TIE1 overexpression

*TIE1* full-length cDNA (OriGene) was amplified by PCR with primers which include EcoRV restriction sites (Fo: cga**gatatc**gtctggcgggtgccccctttc; Re: cgt**gatatc**tcaggcctcctcagctgtggcatc). PCR product was loaded on agarose gel and the corresponding band was purified using Wizard SV kit (Promega, Madison, WI, USA) following the manufacturer’s instructions. EcoRV restriction enzyme was used to digest PCR product and pCCL-6HF vector [64], then *TIE1* was subcloned into pCCL-6HF vector [64]. The pCCL-6HF-TIE1 construct was verified by DNA sequencing. 293T cells were transfected with a mix of 1/3 ΔHelper, 1/3 VsVg and 1/3 pCCL-6HF-TIE1 construct using Lipofectamine reagent 3000 (Invitrogen) following the manufacturer’s instructions. The day after, the supernatant of transfected 293T containing lentiviral particles was added in a six-well plate containing 1 × 10^5^ endothelial cells for 72 h. Infected cells were seeded in 25-cm^2^ flasks to grow and three clones were selected. The TIE1 overexpression was verified by immunofluorescence and western blot (Supplementary Fig. 3).

### Immunocytochemistry

In total, 2 × 10^4^ endothelial cells were seeded on a sterile coverslip in a 12-wells plate. The next day, cells were washed twice with PBS, fixed with paraformaldehyde 4% for 10 min, washed twice and incubated with PBS1X NH_4_CL 50 mM for 10 min, each at room temperature. After washing with PBS1X, cells were permeabilized with PBS1X Triton 0.2% for 10 min and after two washed cells were blocked in PBS1X FBS 5% (HyClone) for 1 h, each at room temperature. Cells were probed with TIE1-PE antibody (R*&*D Systems) for 2 h at room temperature. After wash, coverslips were mounted in ProLong^®^ Gold Antifade reagent (Invitrogen) and observed with a Nikon Eclipse 90*i* microscope.

### Co-immunoprecipitation

Cells were lysed in lysis buffer supplemented with an inhibitor cocktail. Equal amounts of protein were incubated with anti-TIE2 antibody (R*&*D Systems) for 2 h in agitation in a cold room. Recombinant Protein G agarose (Invitrogen) was added in each tube for 1 h in a cold room. After centrifugation, beads were washed twice with lysis buffer and once with PBS1X. A mix of Sample Reducing agent and LDS sample buffer 1× (Invitrogen) was added on beads and heated for 4 min at 95 °C. Each sample was submitted to western blot.

### FACS analysis

In total, 5 × 10^5^ endothelial cells were seeded in PBS1X-BSA1% in a 96-well plate. Cells were washed three times with PBS1X-BSA1% at 4 °C and incubated with anti-CD31 FITC antibody (BD Pharmingen^TM^) or anti-TIE1-PE antibody or anti-TIE2 APC antibody (R*&*D Systems) for 1 h in agitation in a dark cold room. Cells were washed twice and suspended with PBS and analysed by MACSQuant^®^ VYB flow cytometers (Miltenyi Biotech). Data were treated with Flow Jo software.

### Mass spectrometry analysis

In total, 1 × 10^6^ HMVECs were seeded into 100-mm dishes. Twenty-four hours later, cells were washed twice with PBS and treated with human recombinant TNF-α (100 ng/ml; Sigma) for 24 or 48 h. After the indicated time, 10 ml of each supernatant was collected and cells debris were removed by centrifugation (4000×*g*, 20 mn). Cleared supernatant was then dialysed against 100 mM ammonium bicarbonate using Amicon^®^ Ultra 15-ml Centrifugal Filters (cut-off 3 kda, Millipore) and concentrated until 2 ml. In total, 10% of the total volume was dried using a vacuum concentrator and kept apart for SDS-PAGE analysis (silver-stained gel for quality check of the sample). The remaining 90% was dried and protein pellets were solubilized in 8 M urea. After cysteine reduction in presence of 10 mM DTT and alkylation with 50 mM iodoacetamide, proteins were digested with high-sequencing-grade trypsin (Promega, Madison, WI). Peptides were further desalted, cleaned on Sep-Pak C18 Plus cartridge according to the manufacturer instructions (Waters, Milford, MA) and were dried down in a centrifugal vacuum system. Samples were reconstituted with 0.1% trifluoroacetic acid in 4% acetonitrile and analysed by liquid chromatography (LC)-tandem mass spectrometry (MS/MS) using an Orbitrap Fusion Lumos Tribrid Mass Spectrometer (Thermo Electron, Bremen, Germany) online with an Ultimate 3000RSLCnano chromatography system (Thermo Fisher Scientific, Sunnyvale, CA). Peptides were separated on a Dionex Acclaim PepMap RSLC C18 column. First peptides were concentrated and purified on a pre-column from Dionex (C18 PepMap100, 2 cm × 100 µm I.D, 100 Å of pore size, 5 µm of particle size) in solvent A (0.1% formic acid in 2% acetonitrile). In the second step, peptides were separated on a reverse-phase LC EASY-Spray C18 column from Dionex (PepMap RSLC C18, 50 cm × 75 µm I.D, 100 Å of pore size, 2 µm of particle size) at 300 nL/min flow rate. After column equilibration using 4% of solvent B (20% water–80% acetonitrile–0.1% formic acid), peptides were eluted from the analytical column by a two steps linear-gradient (4–20% acetonitrile/H_2_O; 0.1 % formic acid for 90 min and 20–45% acetonitrile/H_2_O; 0.1% formic acid for 30 min). For peptide ionisation in the EASY-Spray nanosource, spray voltage was set at 2.2 kV and the capillary temperature at 275 °C. The Orbitrap Lumos was used in data-dependent mode to switch consistently between MS and MS/MS. The time between Masters Scans was set to 3 s. MS spectra were acquired with the Orbitrap in the range of *m/z* 400–1600 at a FWHM resolution of 120,000 measured at 400 *m/z*. AGC target was set at 4.0 e5 with a 50 ms of maximum injection time. For internal mass calibration, the 445.120025 ions were used as lock mass. The more abundant precursor ions were selected and collision-induced dissociation fragmentation was performed in the ion trap to have maximum sensitivity and yield a maximum amount of MS/MS data. The number of precursor ions was automatically defined along run in 3 s windows using the “Inject Ions for All Available parallelizable time option” with a maximum injection time of 300 ms. The signal threshold for an MS/MS event was set to 5000 counts. Charge state screening was enabled to exclude precursors with 0 and 1 charge states. Dynamic exclusion was enabled with a repeat count of 1 and a duration of 60 s.

### Protein identification and quantification

Relative intensity-based label-free quantification (LFQ) and intensity-based absolute quantification (iBAQ) was processed using the MaxLFQ algorithm [61] from the freely available MaxQuant computational proteomics platform, version 1.5.3.8. [65, 66]. The acquired raw LC Orbitrap MS data were first processed using the integrated Andromeda search engine [67]. Spectra were searched against a UniProt *Human* database (date 2017.05; 20200 entries). This database was supplemented with a set of 245 frequently observed contaminants. The following parameters were used for searches: (i) trypsin allowing cleavage before proline; (ii) two missed cleavages were allowed; (ii) monoisotopic precursor tolerance of 20 ppm in the first search used for recalibration, followed by 4.5 ppm for the main search and 0.5 Da for fragment ions from MS/MS; (iii) cysteine carbamidomethylation (+57.02146) as a fixed modification and methionine oxidation (+15.99491) and N-terminal acetylation (+42.0106) as variable modifications; (iv) a maximum of five modifications per peptide allowed and (v) minimum peptide length was seven amino acids and a maximum mass of 4600 Da. The match between runs option was enabled to transfer identifications across different LC-MS/MS based on their masses and retention time within a match time window of 0.7 min and using an alignment time window of 20 min. The quantification was performed using a minimum ratio count of 1 (unique + razor) and the second peptide option to allow the identification of two co-fragmented co-eluting peptides with similar masses. The false discovery rate (FDR) at the peptide and protein levels were set to 1% and determined by searching a reverse database. For protein grouping, all proteins that cannot be distinguished based on their identified peptides were assembled into a single entry according to the MaxQuant rules. The statistical analysis was done with the Perseus programme (version 1.5.6.0) from the MaxQuant environment (www.maxquant.org). The LFQ normalised intensities were uploaded from the proteinGroups.txt file. First, proteins marked as a contaminant, reverse hits, and “only identified by site” were discarded. Quantifiable proteins were defined as those detected in at least 100% of samples in at least one condition. Protein LFQ normalised intensities were base 2 logarithmized to obtain a normal distribution. Missing values were replaced using data imputation by randomly selecting from a normal distribution centred on the lower edge of the intensity values that simulate signals of low abundant proteins using default parameters (a downshift of 1.8 standard deviations and a width of 0.3 of the original distribution). In this way, imputation of missing values in the controls allows statistical comparison of protein abundances that are present only in the inhibitors samples. To determine whether a given detected protein was specifically differential a two-sample *t* test were done using permutation-based FDR-controlled at 0.01 and 0.05 and employing 250 permutations. The *P* value was adjusted using a scaling factor s0 with a value of 1 [68]. The mass spectrometry proteomics data, including search results, will be deposited to the ProteomeXchange Consortium (www.proteomexchange.org) [69] *via* the PRIDE partner repository with the dataset identifier PXD014488.

### Patient study

The study was approved by the local ethics committee (approval number 11-61) following patient informed consent. The PaCaOmics study is registered at www.clinicaltrials.gov with registration number NCT01692873. All pancreatic adenocarcinoma (PDAC) xenograft samples were xenografted in immunocompromised mice producing patient-derived xenografts (PDX) samples that have been generated in our laboratory according to Nicolle et al. [70]. Animal experiments were approved by the local ethics committee and performed following the guidelines of our centre (CRCM). RNA was obtained from all PDX. Next-Generation Sequencing (RNA-seq) was performed on these samples using Illumina’s TrueSeq Stranded RNA LT protocol to obtain 100 base paired-end reads. RNA-seq reads were mapped using STAR and SMAP on the human hg19 and mouse mmu38 genomes. RNA sequencing of PDAC xenograft samples accession number E-MTAB-5039. Characteristics of this cohort were recently published [71].

### Animal study

All experimental protocols were carried out in accordance with the nationally approved guidelines for the treatment of laboratory animals. All experimental procedures on animals were approved by the Comité d’éthique de Marseille numéro 14 (C2EA-14). Mice were kept within the Experimental Animal House of the Centre de Cancérologie de Marseille, pôle Luminy (Centre de Recherche en Cancérologie de Marseille). In all, 5 × 10^5^ PK4A cells were injected in orthotopic in 6-weeks-old males *Ink4a/Arf*^*fl/fl*^; *LSL-Kras*^G12D^ mice under isoflurane anaesthesia [induction: 4% (vol/vol) and maintenance: 1.5% (vol/vol)]. The next day, mice were treated with an intraperitoneal injection of either NaCl for control mice or mouse recombinant TNF-α (5 µg; Gibco) every day for 27 days. After mice sacrifice, tumours were fixed in paraformaldehyde 4% and included in paraffin.

### Immunohistochemistry

The label was performed on Automate Discovery XT (Roche Ventana). In all, 5-µm sections of tissue were deparaffinized with Néo Clear (Dako) and two baths of absolute ethanol. Antigenic sites were unmasked with cell conditioning 1 (CC1) pH 8 (Roche). Sections were incubated with an anti-α-SMA antibody (Abcam) 1 h at 37 °C and with an OmniMap anti-rabbit HRP (Multimer; Roche) 16 min for the detection. Sections were incubated in Haematoxylin II (Roche) 16 min and with Bluing reagent (Roche) 4 min. Sections were dehydrated in two baths of absolute ethanol 1 min and xylene substitute Néo Clear (Dako) and mounted in Pertex (Histolab). Sections were scanned and labelling was quantified with CaloPix software.

### Histological analysis

Colorations were performed on automate Coverstainer (Dako). Sections of tissue were deparaffinized with Néo Clear (Dako) and two baths of absolute ethanol. For HES coloration, 5-µm sections were incubated in the following baths: haematoxylin (Dako) 2 min, tap water 1 min, ethanol 70% 1 min, eosin (Dako) 3 min 30 s, ethanol 96% 1 min and saffron 4 min. For Masson’s trichrome stain 3-µm sections were probed using the Masson’s trichrome kit (Ral Diagnostics) following the manufacturer’s instructions. All sections were dehydrated in three baths of absolute ethanol 1 min and xylene substitute Néo Clear (Dako). Sections were mounted in Pertex (Histolab). They were scanned and labelling was quantified with CaloPix software.

### Statistic

Significance of the differences between groups was calculated using a two-tailed Student’s *t* test. Values are given as mean ± s.d.

## Supplementary information

Supplementary Information
