# Supplementary material for: TNF-α induces endothelial–mesenchymal transition promoting stromal development of pancreatic adenocarcinoma
Source: Cell Death Dis. 2021 Jun 25;12(7):649. doi: 10.1038/s41419-021-03920-4 (PMC8233393; doi:10.1038/s41419-021-03920-4)
Supplement: Supplementary file 1 — Supplementary Information [file 41419_2021_3920_MOESM1_ESM.pdf]

TNF-α induces endothelial-mesenchymal transition promoting stromal development of pancreatic adenocarcinoma

Marjorie Adjuto-Saccone[1], Philippe Soubeyran[1], Julie Garcia[1], Stéphane Audebert[2], Luc Camoin[2], Marion Rubis[1], Julie Roques[1], Bernard Binétruy[3], Juan Lucio Iovanna[1] and Roselyne Tournaire[1]

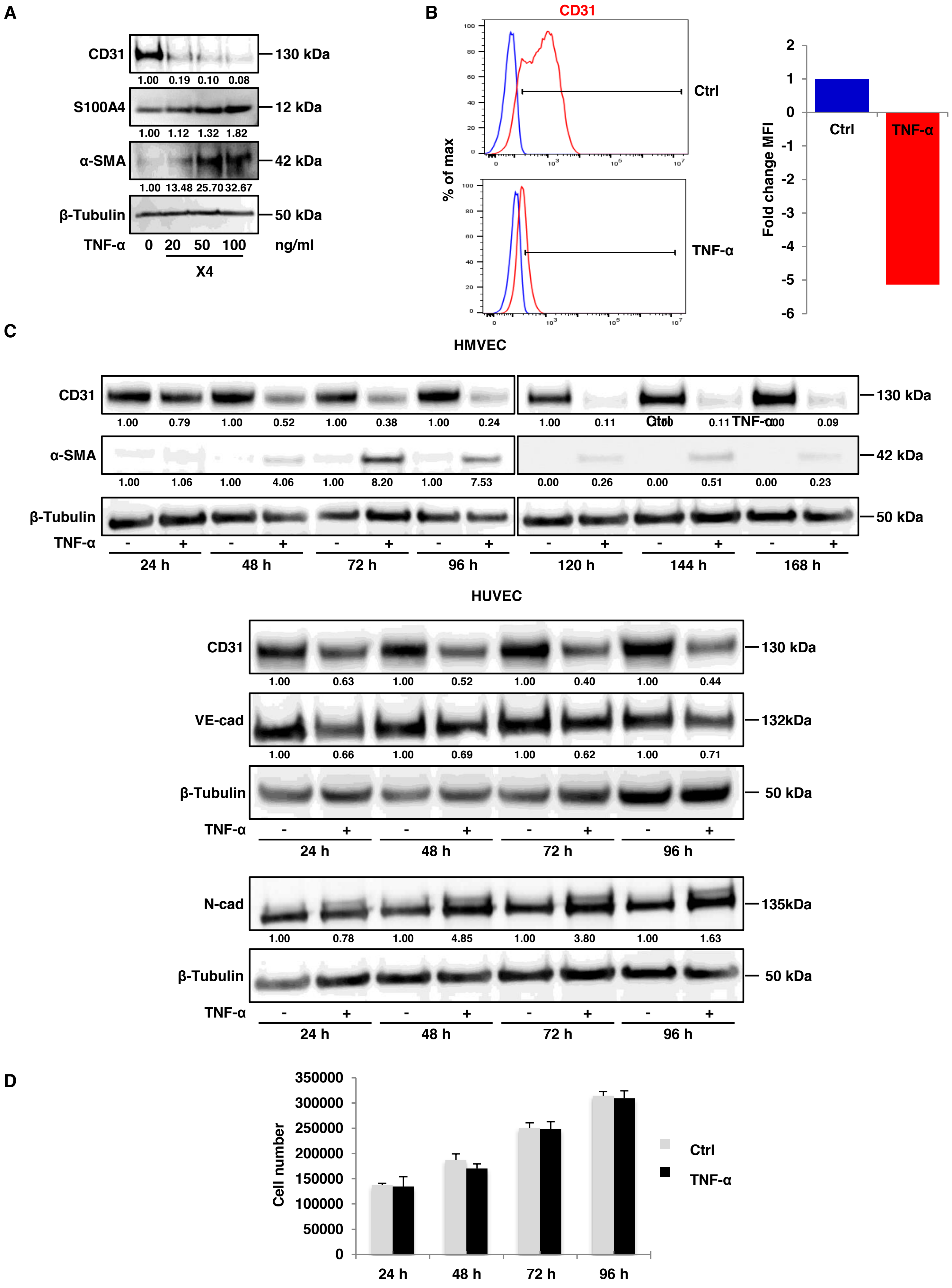
A
CD31
130 kDa
1.00 0.19 0.10 0.08
S100A4
12 kDa
1.00 1.12 1.32 1.82
α-SMA
42 kDa
1.00 13.48 25.70 32.67
β-Tubulin
50 kDa
TNF-α 0 20 50 100 ng/ml
X4
B
CD31
% of max
Ctrl
TNF-α
Fold change MFI
Ctrl
TNF-α
C
HMVEC
CD31
130 kDa
1.00 0.79 1.00 0.52 1.00 0.38 1.00 0.24 1.00 0.11 0.11 0.09
α-SMA
42 kDa
1.00 1.06 1.00 4.06 1.00 8.20 1.00 7.53 0.00 0.26 0.00 0.51 0.00 0.23
β-Tubulin
50 kDa
TNF-α - + - + - + - + - + - + - +
24 h 48 h 72 h 96 h 120 h 144 h 168 h
HUVEC
CD31
130 kDa
1.00 0.63 1.00 0.52 1.00 0.40 1.00 0.44
VE-cad
132kDa
1.00 0.66 1.00 0.69 1.00 0.62 1.00 0.71
β-Tubulin
50 kDa
TNF-α - + - + - + - +
24 h 48 h 72 h 96 h
N-cad
135kDa
1.00 0.78 1.00 4.85 1.00 3.80 1.00 1.63
β-Tubulin
50 kDa
TNF-α - + - + - + - +
24 h 48 h 72 h 96 h
D
Cell number
350000
300000
250000
200000
150000
100000
50000
0
24 h 48 h 72 h 96 h
Ctrl
TNF-α

Supplemental Figure 1. TNF-α induces endothelial-mesenchymal-transition. A, TNF-α decreases the protein expression of vascular endothelial marker (CD31) and increases the protein expression of mesenchymal markers (S100A4, α-SMA). HMVECs were treated every day during 96 h with 20, 50 or 100 ng/ml of TNF-α. Protein expressions were analysed by western blot. **B**, TNF-α decreases the CD31 protein cell surface expression by FACS analysis; Plots represent the % Max of stained cells (red histogram) versus the corresponding isotype control (blue histogram) ; the fold change (FC) in the CD31 MFI is represented. HMVECs were treated for 96 h with 100 ng/ml of TNF-α. **C**, Effect of TNF-α on EndMT in HMVECs (upper panel) and in HUVECs (lower panel) after different incubation times. TNF-α decreases the protein expression of vascular endothelial marker CD31 and increases the protein expression of mesenchymal marker α-SMA. HMVECs and HUVECs were treated for various times with 100 ng/ml of TNF-α. **D**, Effect of TNF-α on HMVECs proliferation. HMVECs were treated for various times with 100 ng/ml of TNF-α. We counted the number of cells by Cell Viability Analyser, Vi-CELL XR. β-tubulin was used as control for western blot analysis. Data are representative of 3 independent experiments. HMVEC, Human Microvascular Endothelial Cell; HUVEC, Human Umbilical Vein Endothelial Cell; α-SMA, α-smooth muscle actin; TNF-α, Tumour Necrosis Factor-α; Ctrl, control; MFI, Mean Fluorescence Intensity.

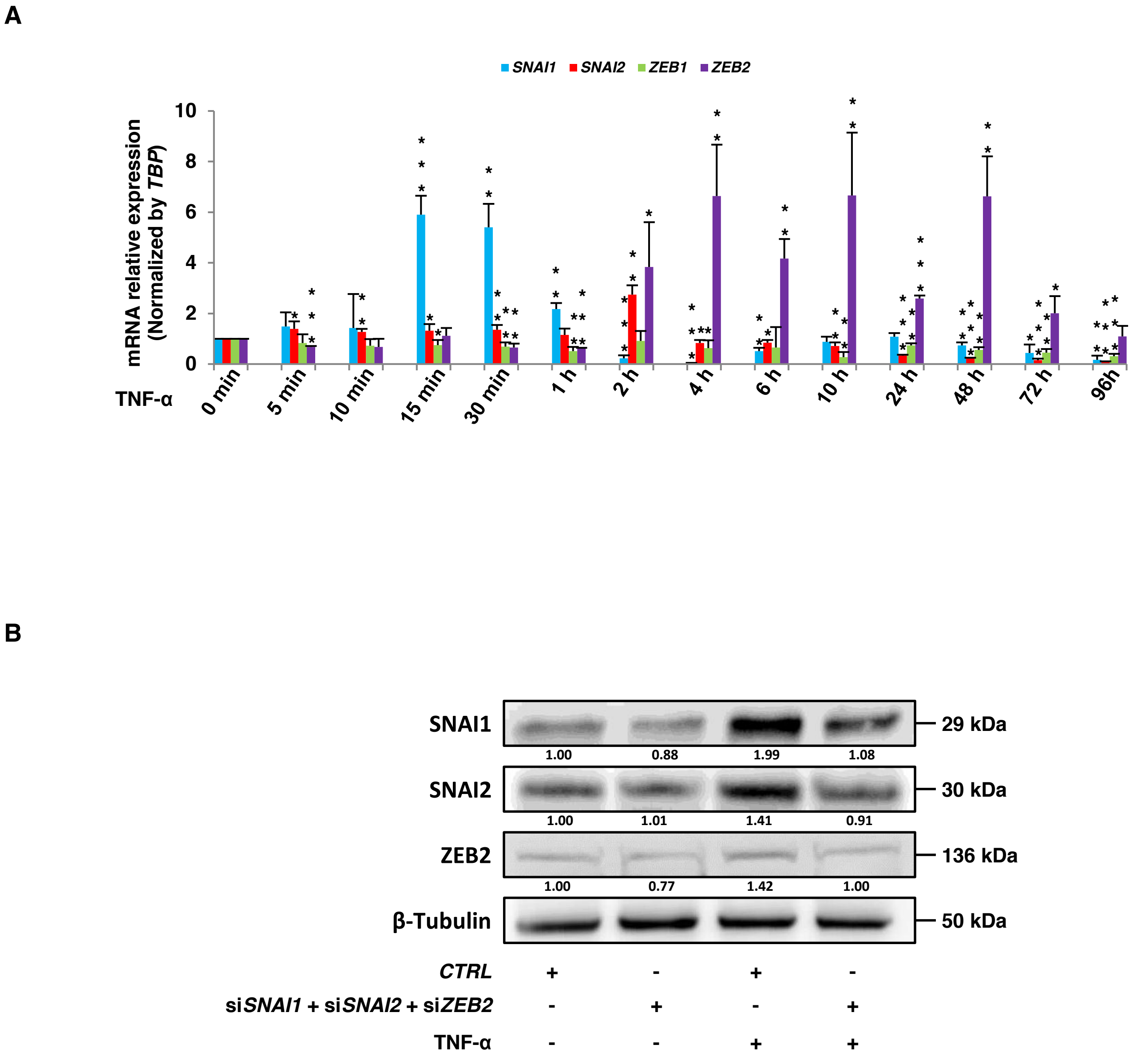
A
SNAI1 SNAI2 ZEB1 ZEB2
mRNA relative expression (Normalized by TBP)
10
8
6
4
2
0
TNF-α
0 min
5 min
10 min
15 min
30 min
1 h
2 h
4 h
6 h
10 h
24 h
48 h
72 h
96h
B
SNAI1
1.00 0.88 1.99 1.08
29 kDa
SNAI2
1.00 1.01 1.41 0.91
30 kDa
ZEB2
1.00 0.77 1.42 1.00
136 kDa
β-Tubulin
50 kDa
CTRL + - + -
siSNAI1 + siSNAI2 + siZEB2 - + - +
TNF-α - - + +

Supplemental Figure 2. Effect of TNF-α on transcription factors. **A**, TNF-α increases the mRNA expression of *SNAI1*, *SNAI2* and *ZEB2*. HMVECs were treated for various times with 100 ng/ml of TNF-α and mRNA level was quantified by RT-qPCR. **B**, *SNAI1*, *SNAI2* and *ZEB2* siRNA suppresses protein expression induced by TNF-α. HMVECs were transfected twice with *SNAI1*, *SNAI2* and *ZEB2* or Ctrl siRNA and treated for 72 h with 100 ng/ml of TNF-α. Protein expressions were quantified by western blot. *TBP* for RT-qPCR and β-tubulin for western blot analysis were used as controls. RT-qPCR histograms show the mean of 3 independent biological experiments and Western blots are representative of 3 independent biological experiments. Significant differences are indicated by solid lines (*P<0.1, **P<0.05, ***P<0.005 by *t*-test). HMVEC, Human Microvascular Endothelial Cell; TNF-α, Tumour Necrosis Factor-α; *CTRL*, control; mRNA, messenger RNA; *TBP*, TATA-box binding protein; siRNA, short-interfering RNA.

A

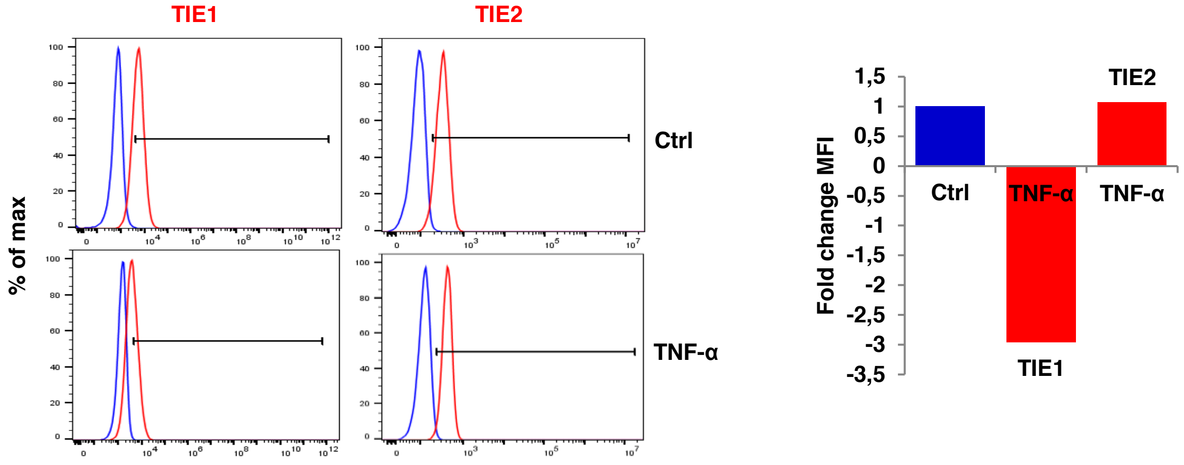
TIE1
TIE2
% of max
Ctrl
TNF-α
Fold change MFI
1,5
1
0,5
0
-0,5
-1
-1,5
-2
-2,5
-3
-3,5
Ctrl
TNF-α
TNF-α
TIE2
TIE1

B

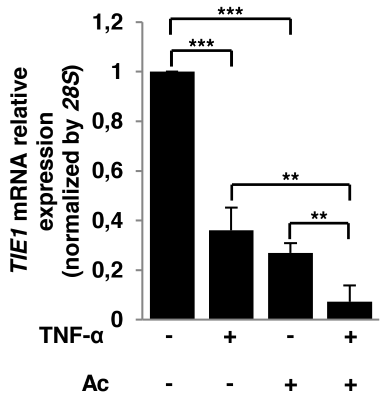
TIE1 mRNA relative expression (normalized by 28S)
1,2
1
0,8
0,6
0,4
0,2
0
***
***
**
**
TNF-α - + - +
Ac - - + +

C

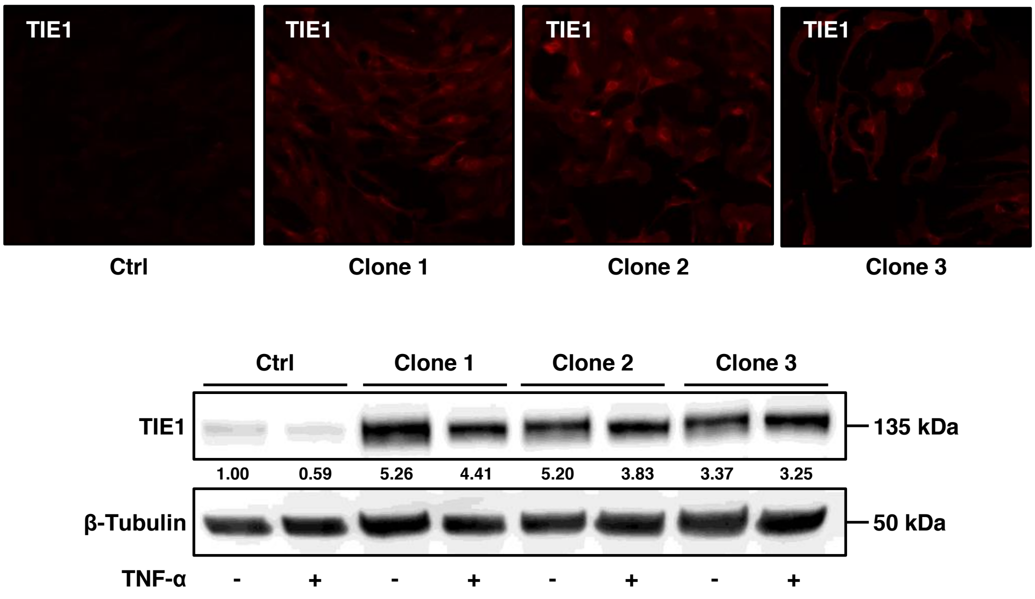
TIE1
TIE1
TIE1
TIE1
Ctrl
Clone 1
Clone 2
Clone 3
Ctrl
Clone 1
Clone 2
Clone 3
TIE1
135 kDa
1.00 0.59 5.26 4.41 5.20 3.83 3.37 3.25
β-Tubulin
50 kDa
TNF-α - + - + - + - +

Supplemental Figure 3. Effect of TNF-α on TIE1 and TIE2 receptors. A, TNF-α decreases the TIE1 protein expression and had no effect on TIE2 expression in FACS analysis. HMVECs were treated for 96 h with 100 ng/ml of TNF-α. Plots represent the % Max of stained cells (red histogram) versus the corresponding isotype control (blue histogram); the fold change (FC) in the TIE1 and TIE2 MFI are represented. **B**, TNF-α acts on *TIE1* mRNA degradation. HMVECs were treated for 2 h with actinomycin D and then for 24 h with 100 ng/ml of TNF-α. mRNA level was quantified by RT-q PCR. RT-qPCR histograms show the mean of 3 independent biological experiments. **C**, TIE1 is overexpresses in three different clones of HMVECs (upper panel) in presence of TNF-α (lower panel). HMVECs were infected with *TIE1*-encoding lentivirus, three infected clones were selected, amplified and treated for 72 h with 100 ng/ml of TNF-α. Protein expressions were quantified by western blot. *28S* for RT-qPCR and β-tubulin for western blot analysis were used as controls. Data are representative of 3 independent experiments. Significant differences are indicated by solid lines (**P<0.05, ***P<0.005 by *t*-test). HMVEC, Human Microvascular Endothelial Cell; TNF-α, Tumour Necrosis Factor-α; Ctrl, control; MFI, Mean Fluorescence Intensity; mRNA, messenger RNA.

A

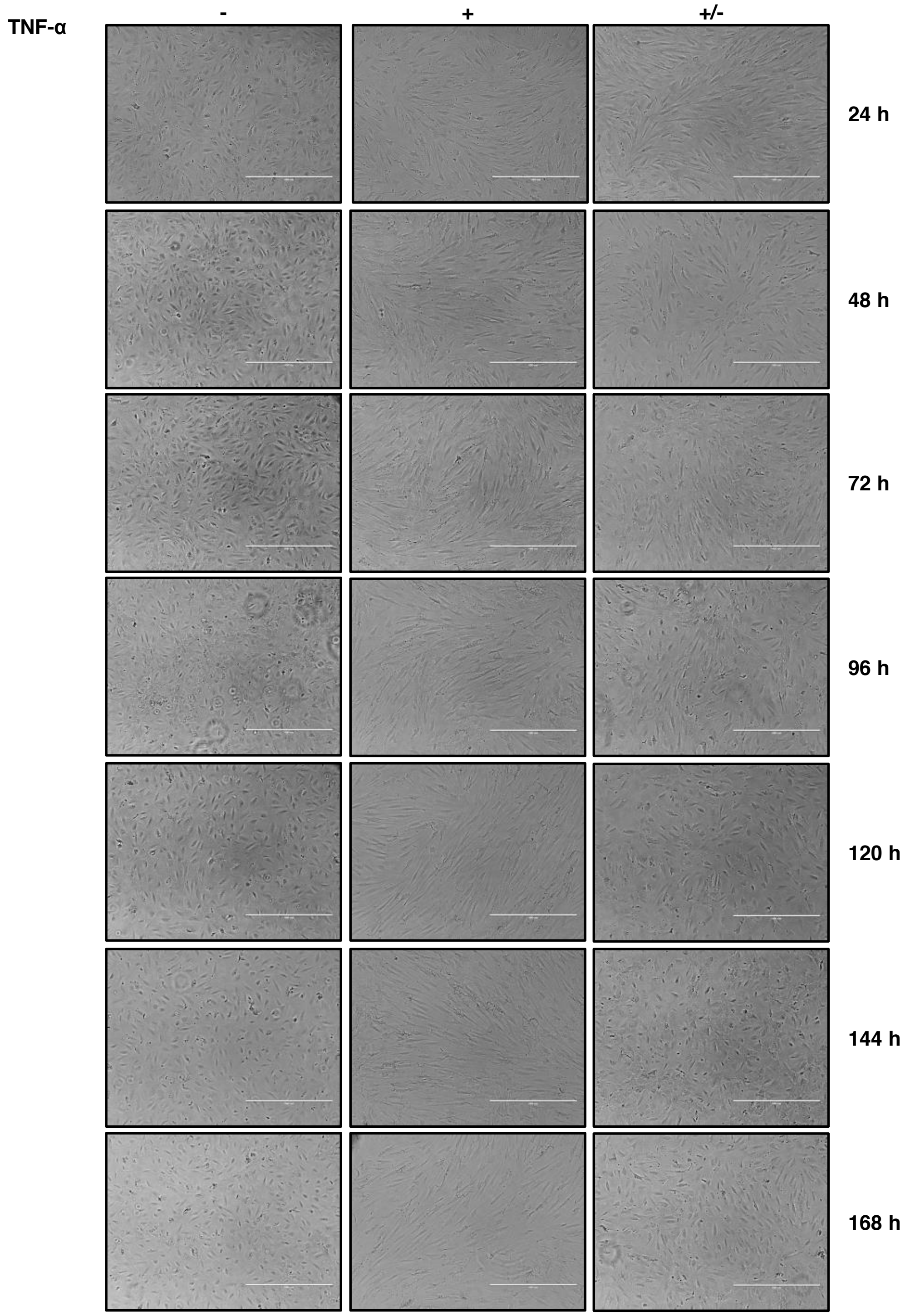
TNF-α
-
+
+/-
24 h
48 h
72 h
96 h
120 h
144 h
168 h

B

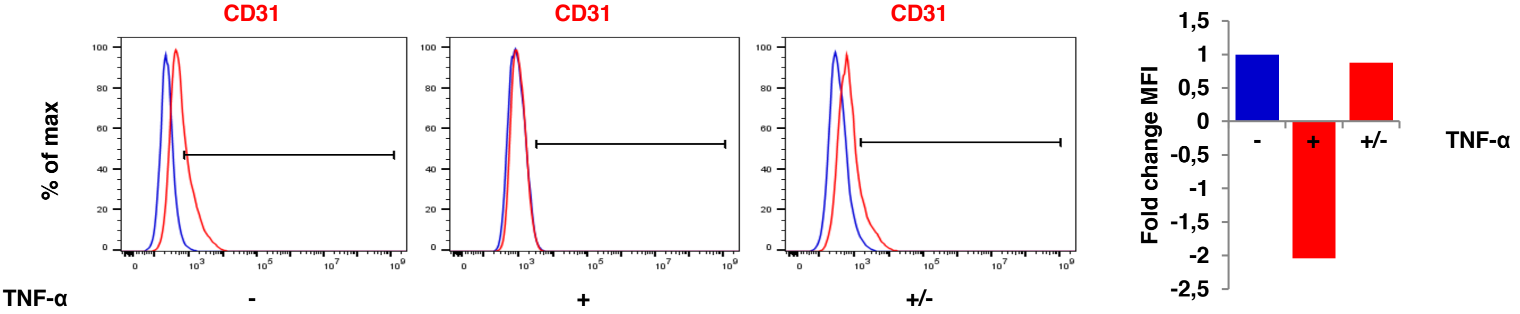
CD31
CD31
CD31
% of max
100
80
60
40
20
0
0
10^3
10^5
10^7
10^9
TNF-α
-
+
+/-
Fold change MFI
1,5
1
0,5
0
-0,5
-1
-1,5
-2
-2,5
-
+
+/-
TNF-α

Supplemental Figure 4. TNF-α-induced EndMT is reversible. A, TNF-α suppression reverses the spindle-shape phenotype induces by TNF-α. **B**, TNF-α reduced the protein expression of vascular endothelial marker CD31 in FACS and 168 h after TNF-α removed, the expression of CD31 is the same as found in TNF-α-free control; plots represent the % Max of stained cells (red histogram) versus the corresponding isotype control (blue histogram) ; the fold change (FC) in the CD31 MFI is represented. HMVECs were treated for 72 h with 100 ng/ml of TNF-α then, cells were washed (-TNF-α) or not (+TNF-α) and cultured from 24 h to 168 h. Scale bars, 400μm. HMVEC, Human Microvascular Endothelial Cell; EndMT, Endothelial-Mesenchymal-Transition ; TNF-α, Tumour Necrosis Factor-α; Ctrl, control; MFI, Mean Fluorescence Intensity.

A

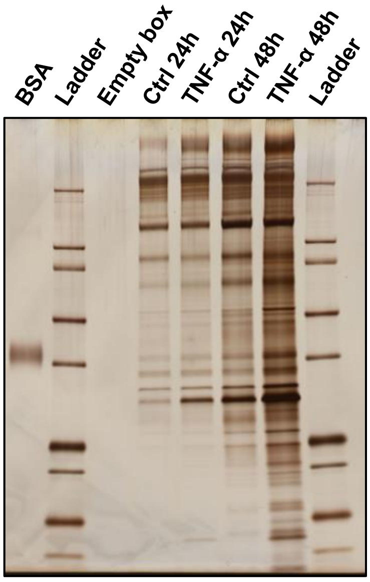

B

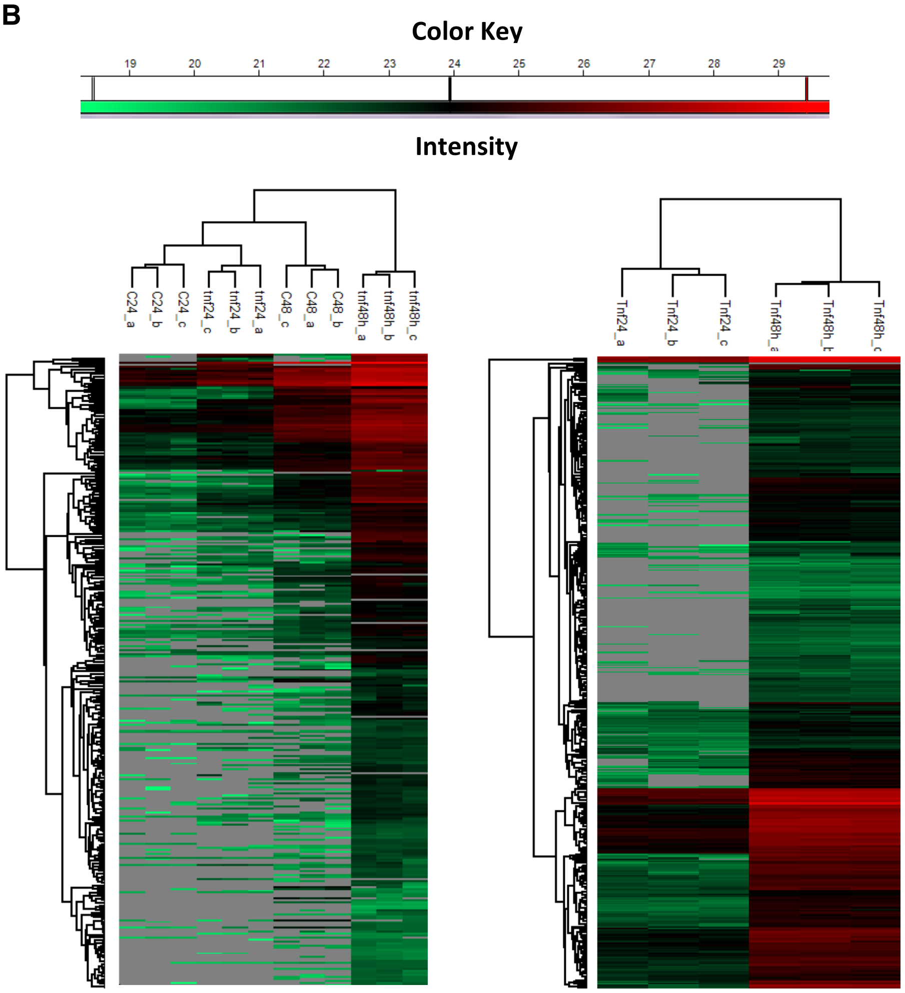

C

Table 1. Summary of proinflammatory proteins

Gene Symbol	Fold Change	p-value
CCL5	836,774	1,88E-04
CSF1	117,49	4,09E-03
ESM1	58,092	1,86E-03
CXCL1	17,803	1,28E-03
IGF2R	17,761	1,99E-03
EDN1	9,313	2,83E-02
STC2	9,217	3,26E-03
SRGN	3,468	7,66E-04
CYR61	2,825	2,41E-04
TGFB2	1,517	1,85E-02

Table 2. Summary of upstream regulators

Upstream Regulator	Activation z-score	p-value
**Inflammation / Fibrose**		
IL1α	3,939	8,17E-14
IL1β	2,689	6,49E-11
IL1RAP	2	1,10E-05
IL1RN	-2,177	1,42E-03
IL12 (complex)	2,354	1,80E-03
IL15	3,939	5,57E-10
IL17A	2,035	4,89E-05
IL18	2,413	1,25E-02
IL27	2,308	1,32E-05
IL32	2,185	2,03E-03
IFNγ	2,295	9,97E-05
STAT1	2,158	1,01E-02
CCL5	2,53	2,40E-07
mir-122	-4,58	4,35E-14
TNFSF12	2,207	2,58E-06
TNFSF14	2,235	2,42E-04
**Signal transduction**		
NFκB (complex)	2,955	2,64E-07
IKBKB	2,219	2,35E-03
RELA	3,221	1,22E-16
JNK	1,921	1,06E-10
MAPK	2	3,07E-03
ERK	2,473	4,96E-05
PI3K (complex)	2,4	4,66E-03

Supplemental Figure 5. Analysis of HMVEC secretome induced by TNF-α. A, TNF-α induces an increase of secreted proteins amount. HMVECs were treated for 24 h and 48 h with 100 ng/ml of TNF-α and supernatants were concentrated and analysed by electrophoresis. The gel was revealed with silver nitrate staining. **B**, Heat map of proteins differentially expressed between Ctrl and TNF-α at 24 h and 48 h (left panel) and between TNF-α 24 h and TNF-α 48 h (right panel). **C**, HMVECs were treated for 48 h with TNF-α. Table S1 depicts the upstream regulator and Table S2 depicts the inflammatory signature of HMVECs treated with TNF-α. HMVECs were treated for 24 h and 48 h with 100 ng/ml of TNF-α and supernatants were analysed by mass spectrometry. Results were obtained with three technical replicates. HMVEC, Human Microvascular Endothelial Cell; TNF-α, Tumour Necrosis Factor-α; Ctrl, control; BSA, Bovin Serum Albumin.

Table 3. siRNA sequences

siRNA	Sequence (5' to 3')	Direction	Lenght
CTRL	CCUACAUCCCGAUCGAUGAUG	Forward	23
CTRL	CAUCAUCGAUCGGGAUGUAGG	Reverse	23
SNAI1	CCACAGAAAUGGCCAUGGGAAGG	Forward	25
SNAI1	CCUUCCCAUGGCCAUUUCUGUGG	Reverse	25
SNAI2	UCCGAAUAUGCAUCUUCAGGGCGCCCA	Forward	29
SNAI2	UGGGCGCCCUGAAGAUGCAUAUUCGGA	Reverse	29
ZEB2	GGACACAGGUUCUGAAACA	Forward	21
ZEB2	UGUUUCAGAACCUGUGUCC	Reverse	21

Table 4. Quantitative RT-PCR primer sequences

Primer	Sequence (5' to 3')	Direction	Lenght
TBP	TGCACAGGAGCCAAGAGTGAA	Forward	21
TBP	CACATCACAGCTCCCCACCA	Reverse	20
TIE1	CACGACCATGACGGCGAAT	Forward	19
TIE1	CGGCAGCCTGATATGCCTG	Reverse	19
TIE2	TGCCACCCTGGTTTTTACGG	Forward	20
TIE2	TTGGAAGCGATCACACATCTC	Reverse	21
CD31	GGAAAAGGCCCCAATACACTT	Forward	21
CD31	TAAAACGCGGTCCTGTTCCTC	Reverse	21
VE-cad	GATCAAGTCAAGCGTGAGTCG	Forward	21
VE-cad	AGCCTCTCAATGGCGAACAC	Reverse	20
CD34	CCAATCTGACCTGAAAAAGC	Forward	20
CD34	CCACCGTTTTCCGTGTAATA	Reverse	20
SMA	GTGTTGCCCCTGAAGAGCAT	Forward	20
SMA	GCTGGGACATTGAAAGTCTCA	Reverse	21
S100A4	GATGAGCAACTTGGACAGCAA	Forward	21
S100A4	CTGGGCTGCTTATCTGGGAAG	Reverse	21
N-cad	GAGGAGTCAGTGAAGGAGTCA	Forward	21
N-cad	GGCAAGTTGATTGGAGGGATG	Reverse	20
COL1A1	GTCTTCTGCAACATGGAGAC	Forward	20
COL1A1	CAGTGGTAGGTGATGTTCTG	Reverse	21
28S	AACGAGATTCCCACTGTCCC	Forward	20
28S	CCGTAAAACGACGGCCAG	Reverse	18
